# DNASE1L3 inhibits proliferation, invasion and metastasis of hepatocellular carcinoma by interacting with β‐catenin to promote its ubiquitin degradation pathway

**DOI:** 10.1111/cpr.13273

**Published:** 2022-06-24

**Authors:** Bo Li, Yu‐Zhen Ge, Wei‐Wei Yan, Bin Gong, Kun Cao, Rui Zhao, Chao Li, Ye‐Wei Zhang, Yi‐Heng Jiang, Shi Zuo

**Affiliations:** ^1^ Department of Clinical Medicine Guizhou Medical University Guiyang Guizhou China; ^2^ Department of Hepatobiliary Surgery The Affiliated Hospital of Guizhou Medical University Guiyang Guizhou China; ^3^ Cancer Center, Integrated Hospital of Traditional Chinese Medicine Southern Medical University Guangzhou China; ^4^ Department of General Surgery The First People's Hospital of Fuquan Fuquan Guizhou China

## Abstract

As a member of the deoxyribonuclease 1 family, DNASE1L3 plays a significant role both inside and outside the cell. However, the role of DNASE1L3 in hepatocellular carcinoma (HCC) and its molecular basis remains to be further investigated. In this study, we report that DNASE1L3 is downregulated in clinical HCC samples and evaluate the relationship between its expression and HCC clinical features. In vivo and in vitro experiments showed that DNASE1L3 negatively regulates the proliferation, invasion and metastasis of HCC cells. Mechanistic studies showed that DNASE1L3 recruits components of the cytoplasmic β‐catenin destruction complex (GSK‐3β and Axin), promotes the ubiquitination degradation of β‐catenin, and inhibits its nuclear transfer, thus, decreasing c‐Myc, P21 and P27 level. Ultimately, cell cycle and EMT signals are restrained. In general, this study provides new insight into the mechanism for HCC and suggests that DNASE1L3 can become a considerable target for HCC.

## Graphical abstract

Decreased expression of DNASE1L3 is associated with poor prognosis in patients with HCC

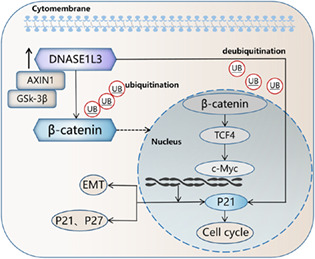

DNASE1L3 inhibits the proliferation and cell cycle of HCC cells in vitro and promotes the invasion and metastasis of HCC cells

DNASE1L3 interacts with β‐catenin and promotes its binding to the β‐catenin destroying complex

DNASE1L3 interacts with P21 and stabilizes P21 by mediating the deubiquitin activity

## 1 INTRODUCTION

Hepatocellular carcinoma (HCC) is one of the most common malignant tumours with the characteristics of invasion and metastasis, and has high morbidity and mortality worldwide.^1^, ^2^ At present, comprehensive treatments such as surgical resection, liver transplantation, radiofrequency ablation and transarterial chemoembolization are the main treatment options for HCC and are only effective for patients with early diagnosis.^3^, ^4^ However, some patients with HCC experienced early recurrence after surgery. In addition, 80% of HCC patients are diagnosed at an advanced stage without particularly effective therapeutic options.^5^, ^6^ Therefore, actively exploring the molecular mechanism of HCC will help to find more effective biomarkers as underlying HCC development and progression.

DNASE1L3, called deoxyribonuclease γ, is a member of the deoxyribonuclease 1 family. It is mainly secreted by macrophages and dendritic cells in the liver and spleen. The protein has DNA hydrolysis activity and can cleave single‐ or double‐stranded DNA, which is an important part of maintaining the human plasma DNA homeostasis.^7^, ^8^, ^9^ Previous studies have revealed that the *DNASE1L3* gene is associated with many diseases, including cancer. DNASE1L3 is correlated with breast cancer signalling, renal clear cell carcinoma staging.^10^, ^11^ Furthermore, DNASE1L3 was found to be a potential prognostic biomarker in lung adenocarcinoma and colon cancer.^12^, ^13^ Recent studies reported that DNASE1L3 could inhibit glycolysis in hepatoma cells and promote the tricarboxylic acid cycle involved in PTPN2‐HK2 and CEBPβ‐p53‐PFK1 pathways, thereby inhibiting HCC progression.^14^, ^15^ Based on various bioinformatic analyses, we found that the role of DNASE1L3 is associated with HCC proliferation, invasion and metastasis, but the underlying molecular mechanisms is unclear.

Ectopic activation of the β‐catenin signalling pathway plays a significant role in the occurrence and development of cancer，including colorectal cancer, liver cancer, lung cancer and so on.^16^, ^17^, ^18^ β‐catenin in the cytoplasm is controlled by β‐catenin destruction complex, including the tumour suppressor axin and adenomatous colonic polyps (APC), Ser/Thr kinase glycogen synthase kinase 3 (GSK‐3), casein kinase 1 (CK1) and the E3‐ubiquitin ligase β‐TrCP. In the absence of Wnt stimulation, the complex is phosphorylated and targeted to β‐catenin for ubiquitin‐proteasomal degradation. When the signal is activated, the complex is degraded resulting in the accumulation of β‐catenin in the cytoplasm and then enter the nucleus，which can interact with the LEF/TCF transcription factors to activate the transcription of target genes related to the Wnt pathway, such as c‐Myc, CyclinD1 and CDKN1A (P21).^19^, ^20^ Many studies have shown that abnormal activation of β‐catenin promotes the proliferation, invasion, metastasis and recurrence of HCC.^21^, ^22^, ^23^ In the subsequent study, Co‐immunoprecipitation (Co‐IP) assays combined with mass spectrometry analysis demonstrated that DNASE1L3 bind to β‐catenin in DNASE1L3‐overexpressing Huh7 cells. However, the relationship between DNASE1L3 and β‐catenin in HCC is unknown.

In this study, DNASE1L3 inhibited HCC proliferation, invasion and metastasis. Mechanistically, DNASE1L3 inhibits HCC progression by interacting with β‐catenin and promoting its ubiquitin degradation pathway. Meanwhile, DNASE1L3 can bind to P21 and promote the deubiquitination degradation of P21.

## 2 MATERIALS AND METHODS

### 2.1 Clinical specimens

After obtaining informed consent, 28 postoperative specimens of liver cancer and the corresponding paracancerous tissues were collected from the Department of Hepatobiliary Surgery, Affiliated Hospital of Guizhou Medical University. In each patient sample, the diagnosis of HCC was confirmed by the tissue pathologist. At the same time, the scheme adopted in this study was approved by the Ethics Review Committee of our hospital.

### 2.2 Cell culture

All cell lines were cultured in a 37°C and 5% CO_2_ incubator supplemented with 10% FBSMagne DMEM medium. Liver cancer cell lines, including Huh7 and HCCLM3, were purchased from the cell bank of the Chinese Academy of Sciences, and LO2 was obtained from the Cancer Institute of Southern Medical University.

### 2.3 Generation and infection of lentivirus

Human *DNASE1L3* gene was inserted into the GV367 vector to construct DNASE1L3 lentivirus (LV‐DNASE1L3) (GeneChem, Shanghai, China). Lentiviruses with an empty GV367 vector (LV‐NC) were constructed as controls (GeneChem, Shanghai, China). According to the manufacturer's scheme, hepatoma cells were infected with LV‐NC or LV‐DNASE1L3, and polyclonal cells with green fluorescent protein signals were selected for further experiments.

### 2.4 Cell transfection

For DNASE1L3 and β‐catenin overexpressing plasmids, the plasmids were designed, synthesized and obtained from GeneChem (Shanghai, China). Aiming at DNASE1L3 siRNA knockdown, siRNA (Table S2) was designed, synthesized and obtained from RiboBio Corporation (Guangzhou, China), and then transient transfection was carried out with Lipofectamine 3000 according to the manufacturer's scheme. After 24–48 h of transfection, the treated cells were tested in the next step.

### 2.5 RNA extraction and RT‐qPCR

The total RNA of cells was extracted by the Cell Total RNA Isolation Kit (Foregene, China), and the total RNA of tissue was extracted by TRIzol reagent (Invitrogen, USA). According to the manufacturer's instructions, TaKaRa's reverse transcription kit was used to transcribe RNA into cDNA. Then, cDNA was used as a template for specific primers for amplification (Table S3). It was used in qPCR through the Bio‐Rad CFX 96 detection system. RT‐PCR, GAPDH were used as the control by Bio‐Rad T100 detection system, and multiple changes in genes were calculated by the 2^−ΔΔCt^ method.

### 2.6 Western blotting analysis

The proteins of tumour tissues and cells were collected from lysis buffer and quantified by the BCA protein assay kit (Thermo Scientific, Waltham, MA, USA). The protein was electrophoresed on SDS polyacrylamide gel and electrotransferred to polyvinylidene fluoride membrane and incubated with the corresponding primary antibody at 4°C for 24 h. The first antibody included DNASE1L3, β‐catenin, c‐Myc, N‐cadherin, E‐cadherin, vimentin, P21, P27, Axin, GSK‐3β, Ubiquitin, GAPDH and β‐tubulin (Table S3). Finally, the chemiluminescence kit purchased from the Thermo Fisher Scientific Company was tested by the ChemiDoc XRS+ molecular imager (Bio‐Rad, Hercules, CA, USA).

### 2.7 Cycloheximide chase assay

Cycloheximide (CHX) (Selleck, Cambridge, UK) was suspended in DMSO (200 mM) before the experiment and stored at −20°C. After the same amount of cells were plated and transferred forward, the concentration was 50 μg/ml and the system was 2 ml of CHX, and then, the cells were incubated at different time gradients. Then, proteins from the cell extract were collected for Western blotting analysis.

### 2.8 Determination of ubiquitin in vivo

DNASE1L3 overexpressing plasmids were transiently transfected into Huh7 and Hep3B cells. After 48 h after transfection, they were co‐cultured with MG132 (20 μg/ml) for 8 h. The protein was extracted by IP lysis buffer. The cell lysates were co‐immunoprecipitated with specific antibodies or IgG, incubated with magnetic beads (protein A/G immunoprecipitation magnetic beads) for 1 h at room temperature, and then washed three times with washing buffer. Finally, after balancing with the protein sample buffer, the protein was released by boiling and a Western blotting analysis was carried out.

### 2.9 Co‐immunoprecipitation

Total protein was extracted from the cell and quantified according to the manufacturer's instructions. A total of 5 mg proteins were shaken and incubated overnight with a 4 μg specific antibody or immunoglobulin G (IgG) at 4°C. After elution, the recovered immune complexes were analyzed by mass spectrometry, Western blotting, and silver staining. Anti‐IgG was used as the negative control.

### 2.10 Determination of CCK‐8 cell viability

Hep3B, Huh7 and LM3 cells were loaded onto a 96‐well plate with 2.5 × 10 ~ 3 cells per well. After the cells were attached, cell viability was evaluated with the cell counting kit‐8 (CCK‐8; DOJINDO, Kumamoto, Japan) at 0, 24, 48 and 72 h. Furthermore, 10 μl CCK‐8 reagent was added into each well and incubated for 2 h. Finally, the absorbance was measured at 450 nm using an enzyme labelling instrument (PerkinElmer EnVision, MA, USA). Each sample was analyzed six times. The experiment was repeated three times.

### 2.11 Colony formation test

Stably transfected cells in the experimental group and the control group were inoculated into 6‐well plates with 1000 cells per well for culture. The culture medium was changed every 48 h. After incubation for 12 days, the colonies were fixed with paraformaldehyde for 15 min and stained with 1% crystal violet for 10 min. The number of colonies containing more than 50 cells was counted under a microscope.

### 2.12 EdU incorporation analysis

According to the manufacturer's instructions, EdU incorporation analysis was carried out through the Apollo 567 In Vitro Imaging Kit purchased from RiboBio Corporation (Guangzhou, China). After co‐culture with EdU solution at a concentration of 1/1000 for 2 h, the cells were fixed with paraformaldehyde (4%) for 30 min, permeated with Triton Xmi 100 (0.5%), and stained with 1x Apollo and 4‐diamino‐2‐phenylindole (DAPI).

### 2.13 Analysis of migration, invasion and wound healing

The ability of cell migration and invasion was measured by a Transwell chamber. The cells were inoculated in the upper chamber of Transwell with or without BDMatrigel at a concentration of 1 × 10^5^ and they were mixed with 100 μl serum‐free DMEM, and DMEM containing FBS (10%) in 500 ml was added to the lower chamber and incubated in a humidified incubator at 37°C and 5% CO_2_ for a corresponding time period. The upper chamber cells were gently removed with wet cotton balls; then 4% paraformaldehyde fixation was performed at the bottom of Transwell for 30 min and 0.1% crystal violet staining was performed for 15 min, and 3 visual fields were randomly selected from each membrane to calculate the average number of invading cells in each sample. The cell plank was grown into a fused monolayer in a six‐hole plate with a density of more than 95%. The tip of a 1‐ml pipette was slid across the cell monolayer to create a linear wound. The process of cell migration was observed by a microscope at 0, 24, and 48 h.

### 2.14 Animal experiment

The animal experiment was performed in accordance with the requirements of the Animal Research Committee of the academic Medical Center of Southern Medical University and the international guidelines for animal care and maintenance. A subcutaneous xenotransplantation mouse model was used to evaluate tumour growth. HCCLM3 cells (5 × 10^6^) were subcutaneously injected into the right abdomen of 3‐week‐old BALB/c male nude mice to evaluate the growth of subcutaneous tumours in the LV‐NC and LV‐DNASE1L3 groups. The tumour size was measured by a Vernier calliper every 3 days. Forty‐five days after inoculation, the tumour was removed, weighed and processed for further experiments.

A mouse model of pulmonary metastasis was established by injecting tumour cells into the tail vein, and HCCLM3 cells (2 × 10^6^) were injected into the tail vein. Fifty days after injection, the small animal in vivo imaging system was used to acquire photographs. First of all, nude mice were placed in an anaesthetic glass box and were anaesthetized with isoflurane. Each nude mouse was then injected intraperitoneally with a preconfigured d‐fluorescein sodium salt (15 mg/ml) at a dose of 200 μl. Nude mice were then placed in the imaging box and the head was placed in an anaesthetized glass mask. The time of injection of d‐fluorescein sodium salt was recorded. When each nude mouse was injected for 10 min, fluorescence imaging was performed according to the operation. Finally, the lung tissue was dissected for further experiments.

### 2.15 Statistical analysis

SPSS25.0 (SPSS, Chicago, USA) was used for statistical analysis. The data were presented as the mean ± SD of at least three independent experiments. Student's *t*‐test or Tukey's multiple comparison test was used for comparison between the two groups, and single‐factor analysis of variance was used for multi‐group comparison. Pearson test detected the correlation between two continuous variables. Survival analysis was carried out by the Kaplan–Meier method and logarithmic rank test. The Cox proportional hazard regression model was used to analyze the independent prognostic factors. The *p* < 0.05 was considered to indicate statistical significance (*p < 0.05, *p* < 0.01, *p* < 0.001).

## 3 RESULTS

### 3.1 DNASE1L3 is downregulated in HCC and associated with poor prognosis

**FIGURE 1:**
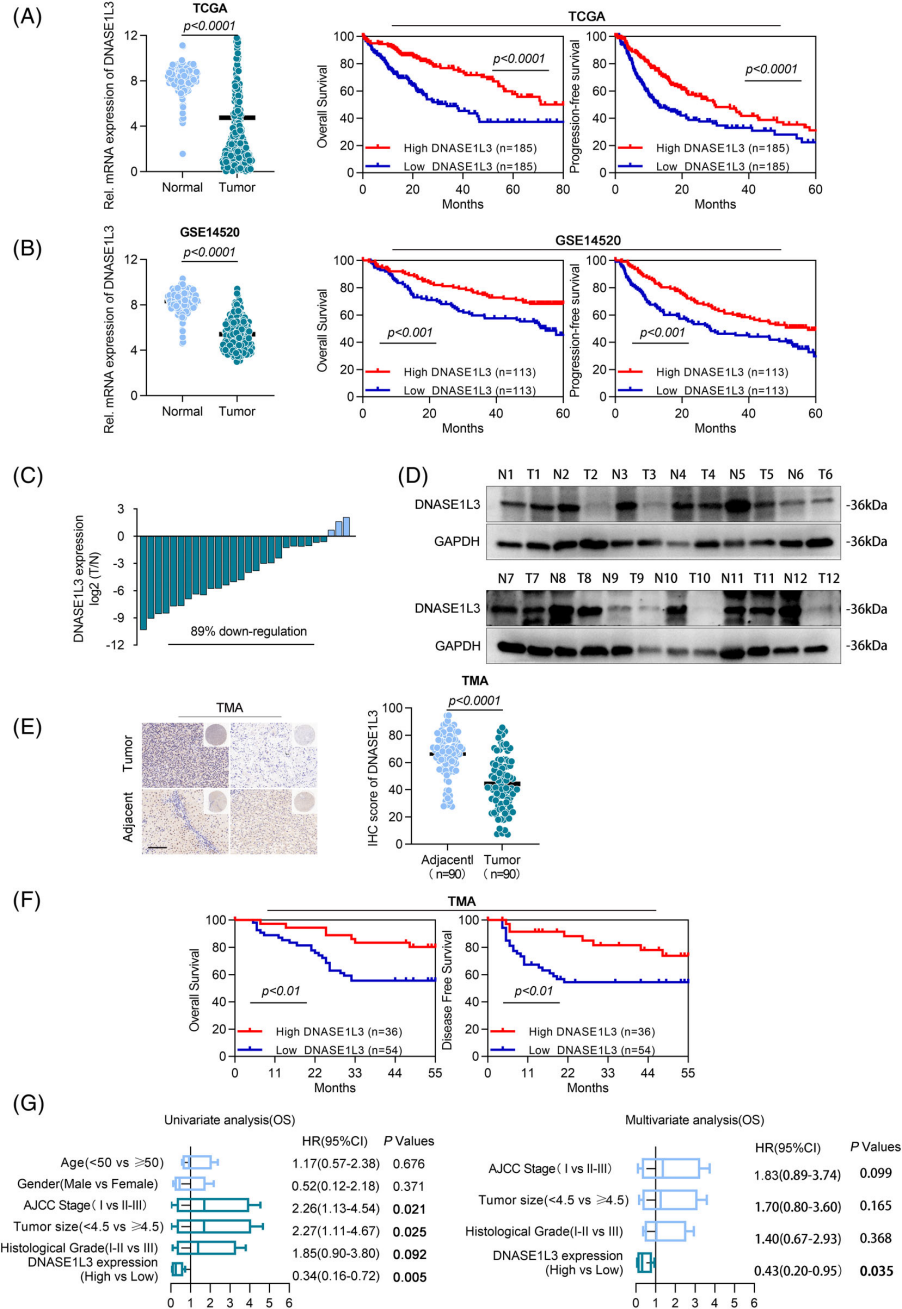
Expression of DNASE1L3 in hepatocellular carcinoma and its prognostic significance. (A and B) The The Cancer Genome Atlas (TCGA) and Gene Expression Omnibus (GEO) database showed that the transcription level of DNASE1L3 was lower in hepatocellular carcinoma (HCC) compared to paracarcinoma tissues, and high expression of DNASE1L3 has a good overall survival (OS) and progression‐free survival (PFS). (C) The transcription level of DNASE1L3 was detected by RT‐qPCR in 28 pairs of HCC tissues and adjacent non‐tumour tissues. The results showed that 25 (89%) of them were significantly down‐regulated. (D) The expression level of DNASE1L3 protein was detected by Western blot in 12 pairs of HCC tissues and adjacent non‐tumour tissues. (E) The expression level of DNASE1L3 in tissue microarray chip was measured by IHC. The expression level of DNASE1L3 in cancer and paracancerous tissues were compared according to the histochemical score of AI intelligence score. Scale bar: 100 μm. (F) Kaplan–Meier analysis showed that patients with high expression of DNASE1L3 had better OS and DFS. (G) Univariate and multivariate analyses of different clinicopathological features in HCC patients.

In order to understand the role of DNASE1L3 in the progression of HCC, we used TCGA (The Cancer Genome Atlas) and GEO (Gene Expression Omnibus) databases to compare the differences of DNASE1L3 mRNA level between tumour samples and normal samples, and to investigate the role of DNASE1L3 in the outcome of HCC patients. The mRNA levels of DNASE1L3 in HCC tissues were significantly lower than that in normal samples, and the overall survival (OS) and progression‐free survival (PFS) of patients with high DNASE1L3 expression were better than those of patients with low DNASE1L3 expression (Figure 1A, B). To further determine the expression of DNASE1L3 in HCC, the level of DNASE1L3 mRNA in 28 pairs of HCC tissues and their matched adjacent non‐tumour tissues were detected by RT‐qPCR, and the protein level of DNASE1L3 in 12 pairs of HCC samples containing adjacent non‐tumour samples were tested by Western Blot. As shown in Figures 1C, D and S1A, the expression of DNASE1L3 was markedly downregulated in HCC. Furthermore, the levels of DNASE1L3 mRNA and protein were decreased in HCC cell lines compared with the immortalized liver cell line LO2 (Figure S1BC). Subsequently, we detected the expression of DNASE1L3 in the tissue microarray containing 99 pairs of HCC and corresponding paracancerous tissues by IHC staining. Consistent with the above results, the expression of DNASE1L3 was significantly down‐regulated in HCC tissues compared with adjacent tissues (Figure 1E). Kaplan–Meier analysis showed that reduced DNASE1L3 levels were correlated with worse OS and disease‐free survival (DFS) in HCC patients (Figure 1F). According to the median value of the AI intelligence score, DNASE1L3 was divided into the high‐ and low‐expression group, and the correlation between DNASE1L3 and clinicopathological features of HCC was further analyzed. DNASE1L3 was negatively correlated with the AJCC stage, tumour size, survival status, and tumour recurrence (Table S1). Meanwhile, univariate and multivariate regression analyses showed that the DNASE1L3 may be a potential independent indicator (Figures 1G and S1D) for OS and DFS in patients with HCC. Taken together, these data indicated the downregulation of DNASE1L3 in HCC and the association between DNASE1L3 and clinicopathology of HCC patients.

### 3.2 DNASE1L3 inhibits the proliferation, invasion and metastasis of HCC cells in vitro

**FIGURE 2:**
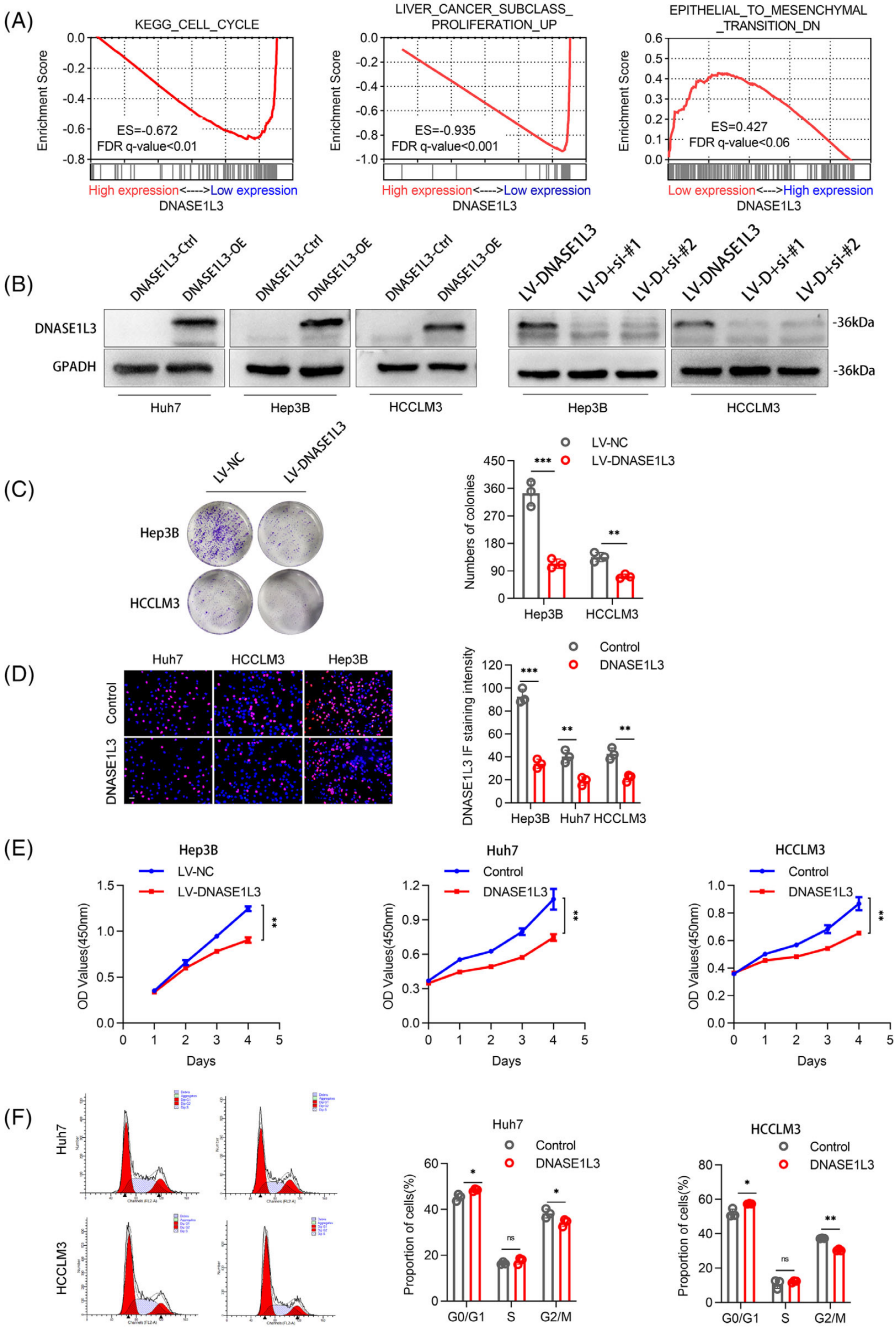
Overexpression of DNASE1L3 inhibits the proliferation of hepatocellular carcinoma cells. Gene enrichment analysis showed that the predefined genomes involved in cell cycle, cell proliferation, cell metastasis and invasion were significantly enriched in low‐level DNASE1L3, indicated that DNASE1L3 may has an inhibitory effect on above properties. (B) Western blot analysis of the expression of DNASE1L3 in hepatocellular carcinoma (HCC) cells transfected with overexpression plasmid or siRNA. (C) Colony formation assays revealed that DNA overexpression inhibited the proliferation of Hep3B and HCCLM3 cells. (D) EdU assay showed that the proliferation of Huh7, HCCLM3 and Hep3B cells decreased after DNASE1L3 overexpression. Scale bar: 200 μm. (E) DNASE1L3 overexpression markedly inhibited cell viability by CCK‐8 assay. (F) Flow cytometry showed that the overexpression of DNASE1L3 increased the G0/G1 phase of Huh7 and HCCLM3 cells and blocked the cell cycle in G1 phase. Student's *t‐* test. Mean ± SD (*p < 0.05; **p < 0.01). LV‐D + si‐#1/LV‐D + si‐#2: overexpression of DNASE1L3 by lentivirus, and then transient knockdown of DNASE1L3 using siRNA#1/siRNA#2.

**FIGURE 3:**
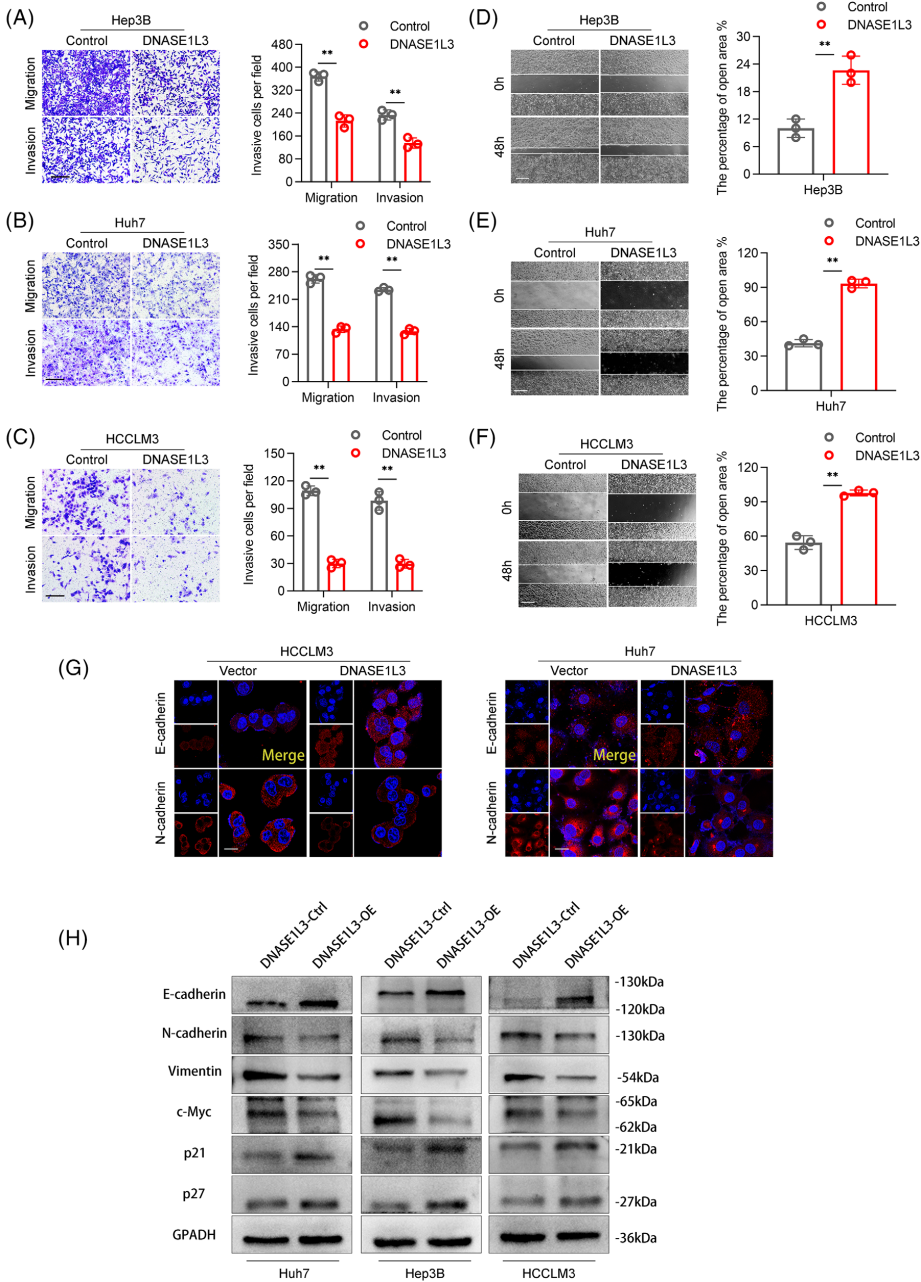
Overexpression of DNASE1L3 inhibits invasion and metastasis of hepatocellular carcinoma cells. (A–C) Transwell assay showed that the metastatic and invasive ability of Huh7, HCCLM3 and Hep3B cells decreased after DNASE1L3 overexpression. Scale bar: 200 μm. (D–F) Wound healing assay showed that the migratory ability of Huh7, HCCLM3 and Hep3B cells decreased after DNASE1L3 overexpression. Scale bar: 200 μm. (G) The relative fluorescence intensities of E‐cadherin and N‐cadherin were assessed by IF staining in Huh7 and HCCLM3 cells after DNASE1L3 overexpression. Scale bar: 20 μm. (H) The expression levels of Vimentin, c‐Myc, N‐cadherin, E‐cadherin, p21 and p27 in HCCLM3 and Hep3B cells were detected by Western blot. Student's *t*‐test. Mean ± SD (*p < 0.05; **p < 0.01).

**FIGURE 4:**
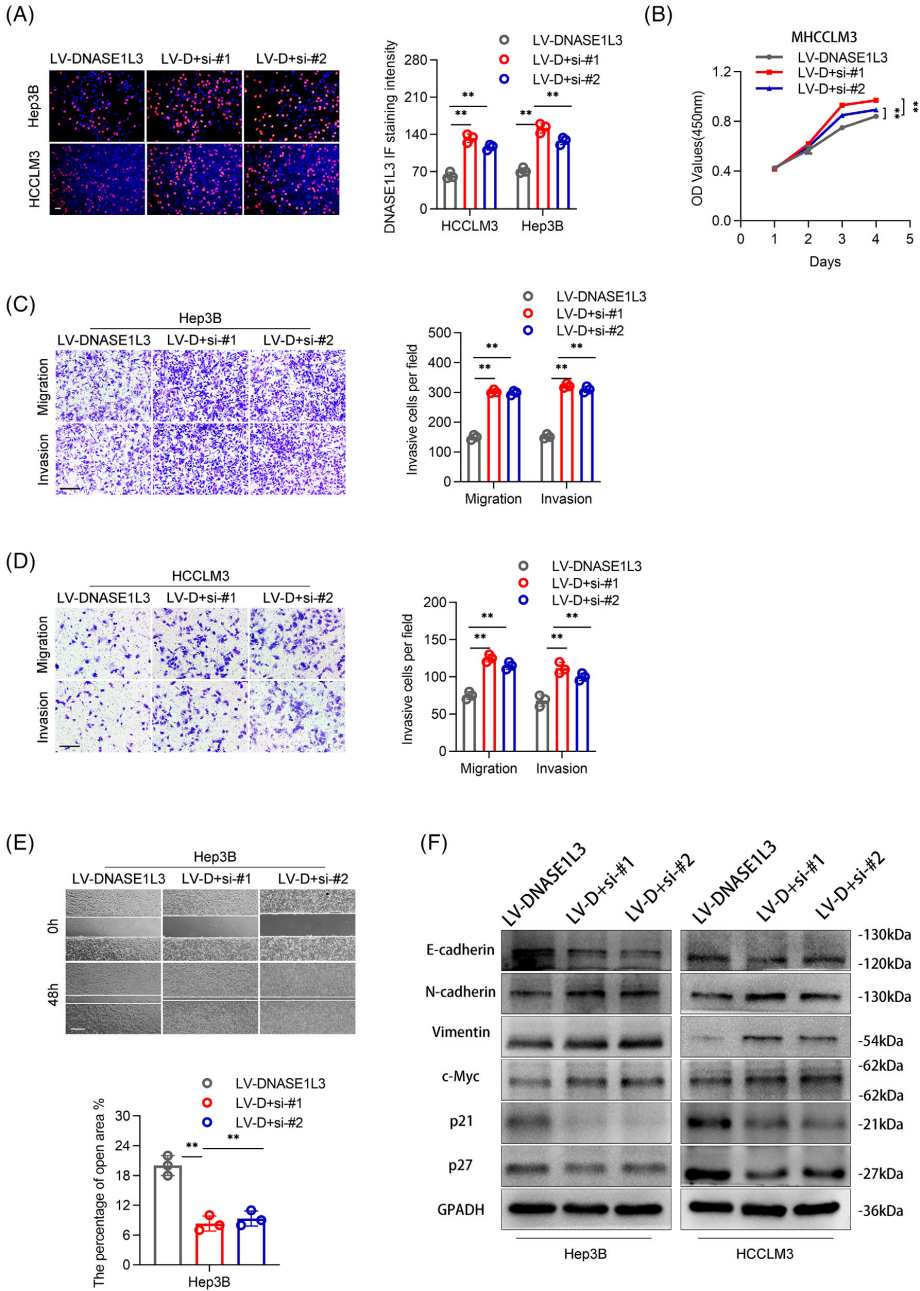
Knockdown of DNASE1L3 promotes proliferation, invasion and metastasis of hepatocellular carcinoma cells. EdU assay showed that the knockdown of DNASE1L3 promoted the proliferation of HCCLM3 and Hep3B cell. Scale bar: 200 μm. (B) CCK‐8 assay revealed that the proliferation of HCCLM3 cell was decreased after DNASE1L3 knockdown, compared with the control. (C and D) Transwell assay demonstrated that knockdown of DNASE1L3 promotes the metastasis and invasion of HCCLM3 and Hep3B cells. Scale bar: 200 μm. (E) Wound healing assay showed that knockdown of DNASE1L3 promotes the migration of Hep3B cells. Scale bar: 200 μm. (F) The expression levels of Vimentin, c‐Myc, N‐cadherin, E‐cadherin, p21 and p27 in Huh7, HCCLM3 and Hep3B cells were detected by Western blot. Student's *t*‐test. Mean ± SD (*p < 0.05; **p < 0.01).

The biological function of DNASE1L3 in HCC was studied by gene enrichment analysis (Gene Set Enrichment Analysis, GSEA). We found that predefined genomes involved in the cell cycle, cell proliferation, cell metastasis and invasion were significantly enriched in low‐level DNASE1L3 (Figure 2A). Based to the low expression of DNASE1L3 in most HCC cell lines, we introduced plasmids or lentivirus into Huh7, Hep3B and HCCLM3 cells to upregulate DNASE1L3. Meanwhile, the small interfering RNA (siRNA) was used to knockdown DNASE1L3 again on the basis of stable overexpression of DNASE1L3, and then detected the corresponding protein (Figures 2B and ure S1E‐G). Colony formation assay, EdU and CCK‐8 assay observed that DNASE1L3 overexpression remarkedly inhibited the viability of Huh7, Hep3B and HCCLM3 cells (Figure 2 C‐E). On the contrary, knockdown of DNASE1L3 enhanced the cell viability of Hep3B and HCCLM3 cells (Figure 4A,B). The effect of DNASE1L3 on the cell cycle was evaluated by flow cytometry. The results showed that overexpression of DNASE1L3 increased the G0/G1 phase of Huh7 and HCCLM3 cells and arrested the cell cycle in the G1 phase (Figure 2F). Transwell assay and wound healing assay were used to explore the effect of DNASE1L3 on the invasion and metastasis of HCC cells. DNASE1L3 overexpression significantly reduced the number of invasive and metastatic cells in Transwell and Boyden chambers and decreased the migratory ability of HCC cells, while DNASE1L3 knockdown showed the opposite trend (Figures 3A–F and 4C–E). Similarly, immunofluorescence analysis showed that DNASE1L3 increased the fluorescence intensity of metastasis markers, such as E‐cadherin and N‐cadherin, in Huh7 and HCCLM3 cells (Figure 3G). In addition, we found that overexpression of DNASE1L3 decreased the level of mesenchymal markers, including N‐cadherin and vimentin, while increased the level of epithelial marker E‐cadherin. Furthermore, Western blot demonstrated that DNASE1L3 overexpression increased the expression of P21 and P27 proteins associated with the G1 phase, while DNASE1L3 knockdown decreased these proteins (Figures 3H and 4F). These results suggest that DNASE1L3 has a vital role in the biological properties of HCC cells.

### 3.3 DNASE1L3 inhibits the tumorigenicity and metastasis of HCC cells in vivo

**FIGURE 5:**
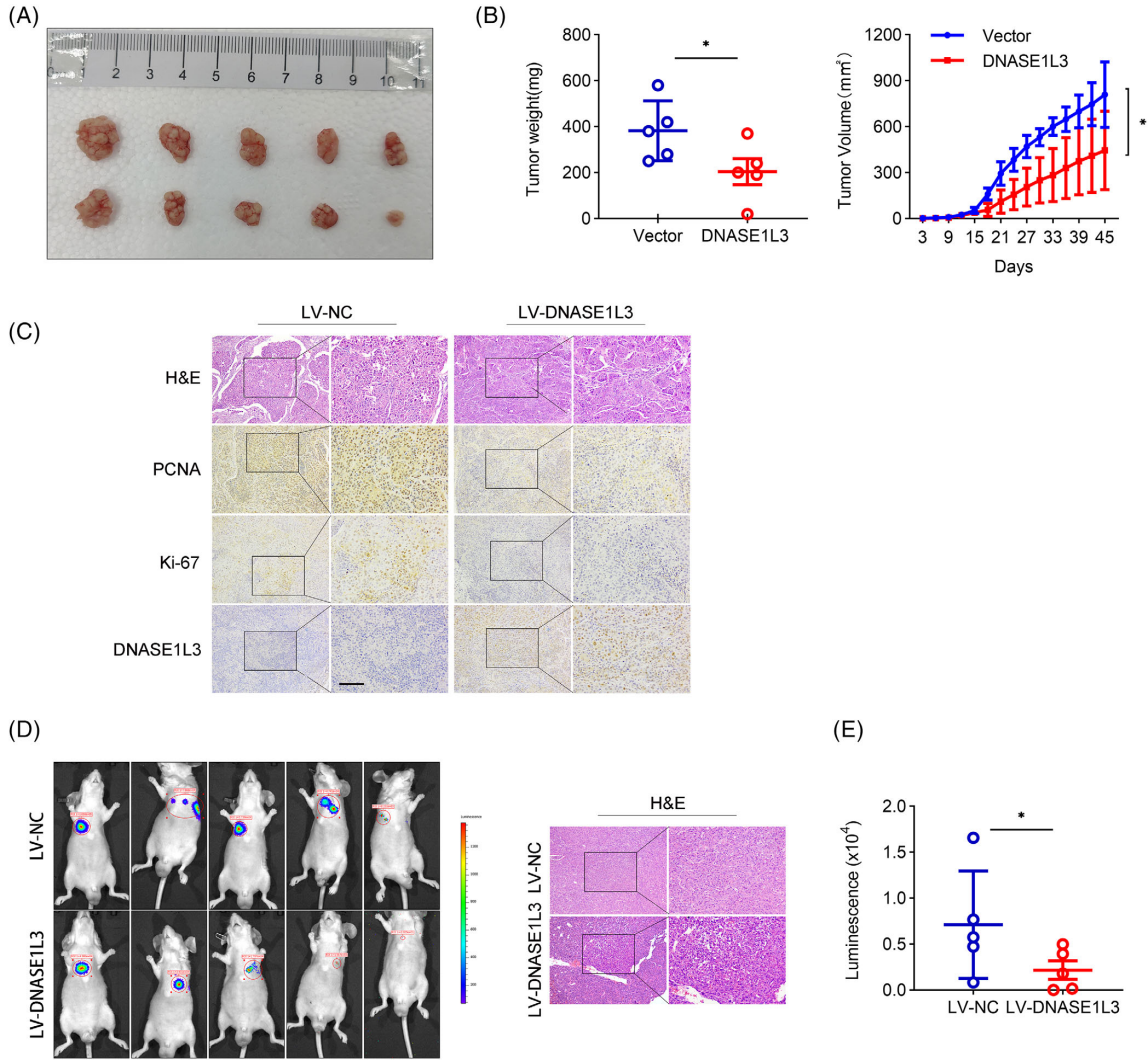
DNASE1L3 inhibits tumorigenicity and metastasis of hepatocellular carcinoma in vivo. Dissected tumours from HCCLM3 cells stably expressing DNASE1L3 and control cells. (B) Nude mice were subcutaneously transplanted with HCCLM3 cells stably expressing DNASE1L3 and control cells, and the growth curve and weight of the tumour were obtained. (C) Representative H&E and IHC staining of dissected tumour tissues were shown. Scale bar: 100 μm. (D) Mouse bioluminescence images derived from the IVIS imaging system and representative images of H&E staining of lung tissue. Scale bar: 100 μm. (E) The lung intensity of LV‐NC and LV‐DNASE1L3 groups was quantified by fluorescence intensity. Student's *t*‐test. Mean ± SD (*p < 0.05; **p < 0.01).

In order to study the biological function of DNASE1L3 in vivo, we established a subcutaneous xenograft tumour model and a lung metastasis model in nude mice. First, the nude mice were divided into two groups, and the DNASE1L3 overexpressing HCCLM3 cells and the control HCCLM3 cells were transplanted to the flanks to generate xenograft tumours, respectively (Figure 5A). Compared with the control group, it was observed that the tumour growth and weight of xenografts were significantly decreased in the DNASE1L3 overexpression group (Figure 5B). IHC staining showed that the expression of DNASE1L3 was increased and the expression of PCNA and Ki‐67 were decreased in the DNASE1L3 overexpression group, compared with the control group. In addition, a lung metastasis model of HCC (Figure 5D) was established by injecting HCCLM3 cells (2 × 10^6^) into the tail vein of nude mice. Compared with the control group, the lung metastasis‐related luminous signal induced by the overexpression of DNASE1L3 was significantly decreased (Figure 5E).

### 3.4 DNASE1L3 binds to β‐catenin and inhibits its activation

**FIGURE 6:**
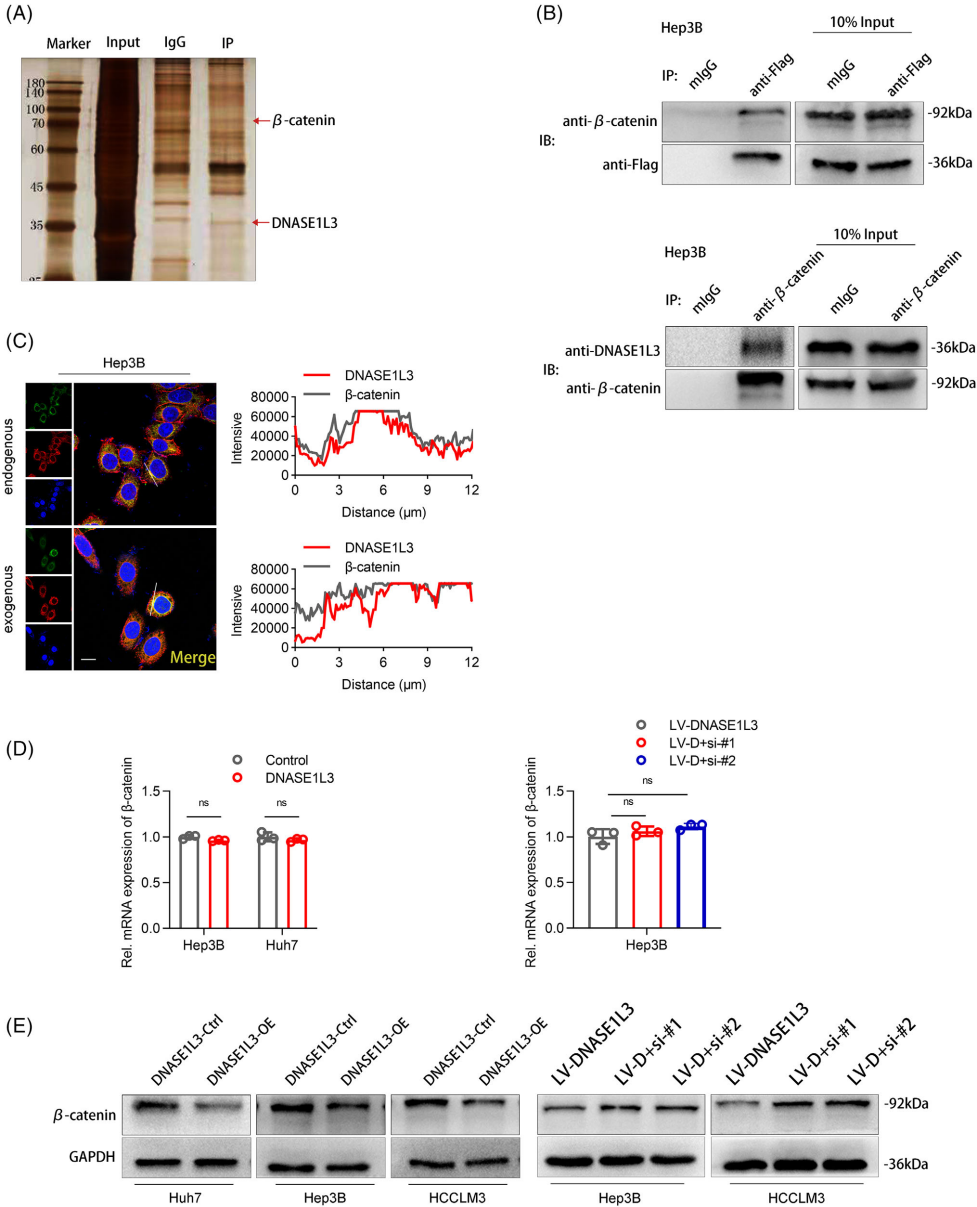
DNASE1L3 binds to β‐catenin and inhibits its activation. Silver staining of SDS‐PAGE gel. IP was performed by the mixture of Huh7 cell lysis. IgG group was used as control. (B and C) The interaction between DNASE1L3 and β‐catenin in Hep3B cells was determined by exogenous and endogenous Co‐IP and cellular immunofluorescence staining. Scale bar: 20 μm. (D) The mRNA level of β‐catenin was detected by RT‐qPCR after DNASE1L3 overexpression or knockdown. (E) The protein expression of β‐catenin was detected by Western blot after DNASE1L3 overexpression or knockdown. Student's *t*‐test. Mean ± SD (*p < 0.05; **p < 0.01).

**FIGURE 7:**
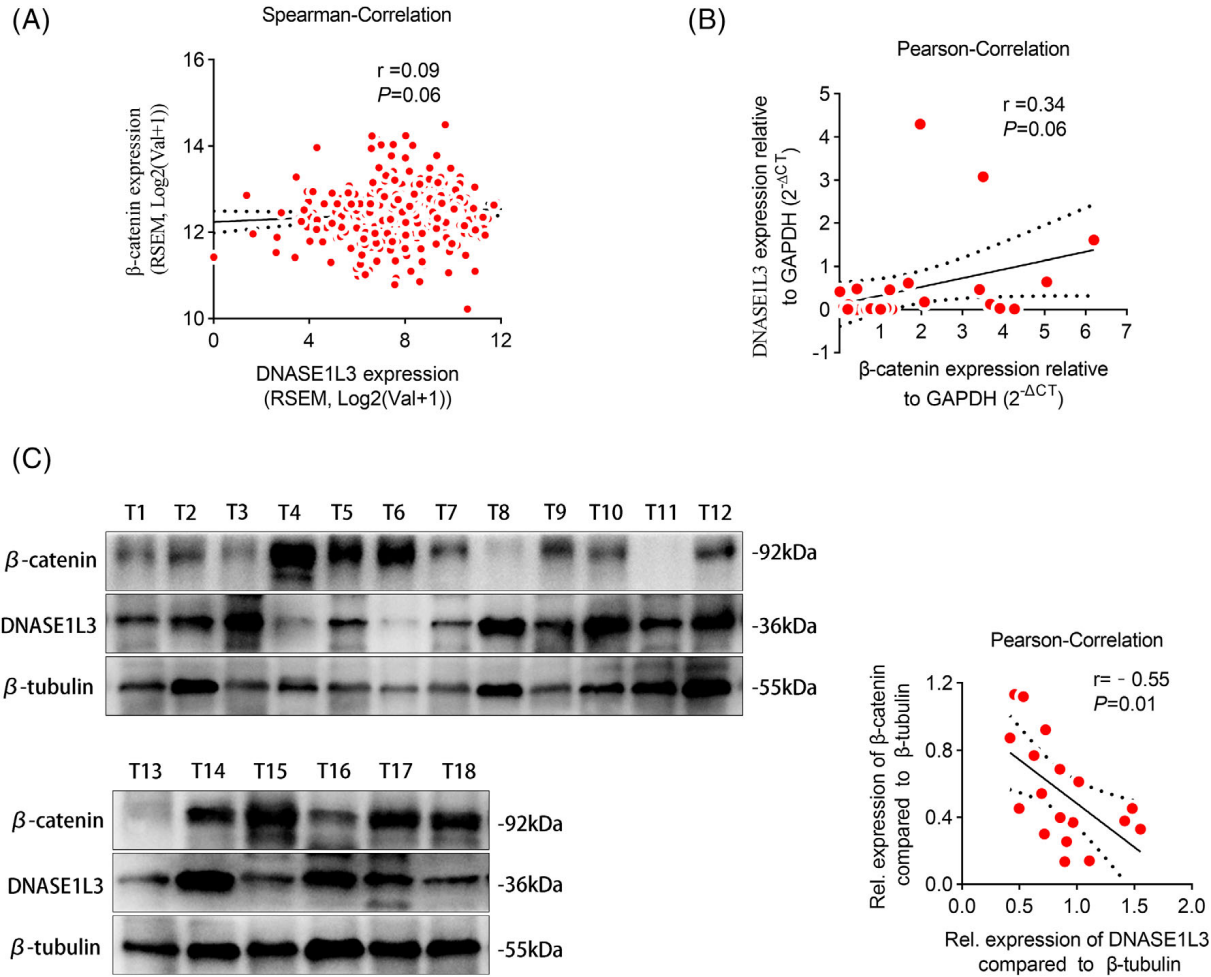
Correlation between DNASE1L3 and β‐catenin. Pearman correlation of DNASE1L3 and β‐catenin transcription levels were analyzed based on RNA‐seq data from TCGA. (B) Pearson correlation of DNASE1L3 and β‐catenin transcription levels were analyzed based on 28 pairs of hepatocellular carcinoma (HCC) tissues. (C) Pearson correlations of DNASE1L3 and β‐catenin protein levels were analyzed based on 18 pairs of HCC tissues. Student's *t*‐test. Mean ± SD (*p < 0.05; **p < 0.01).

The influence of DNASE1L3 on the progression of HCC urges us to further determine its mechanism of inhibition of HCC. Immunoprecipitation (IP) combined with mass spectrometry analysis of DNASE1L3‐interacting complexes in the cell extracts was performed and plenty of novel binding partners including β‐catenin (92 kDa) were identified (Figures 6A and S1H). In order to further prove the interaction between DNASE1L3 and β‐catenin, the exogenous and endogenous Co‐IP assays and cellular immunofluorescence staining were performed (Figure 6B and C). In addition, RT‐qPCR and Western blot were used to explore the functional relationship between DNASE1L3 and β‐catenin. We found that overexpression or knockdown of DNASE1L3 did not affect the level of β‐catenin mRNA (Figure 6D). It is worth noting that DNASE1L3 overexpression decreased the level of β‐catenin protein, while DNASE1L3 knockdown increased the expression of β‐catenin protein (Figure 6E). Importantly, we found the expression of β‐catenin was decreased in a dose‐dependent manner after DNASE1L3 plasmid was introduced into 293 T cells (Figure S2A). Then, the TCGA database analysis showed no correlation between the mRNA levels of DNASE1L3 and β‐catenin in HCC tissue (Figure 7A). Furthermore, the levels of DNASE1L3 mRNA and β‐catenin mRNA in 28 pairs of HCC samples were detected by RT‐qPCR, and found that there was no correlation between DNASE1L3 mRNA and β‐catenin mRNA in HCC (Figure 7B). To further determine the regulatory effect of DNASE1L3 on β‐catenin at the protein level. Western blot was performed using 18 HCC samples, and found that the expression of DNASE1L3 protein was negatively correlated with the expression of β‐catenin protein (Figure 7C). Therefore, we speculate that the effect of DNASE1L3 on β‐catenin is mainly at the post‐transcriptional translation level.

### 3.5 DNASE1L3 recruits β‐catenin destruction complexes and promotes β‐catenin ubiquitination and degradation

**FIGURE 8:**
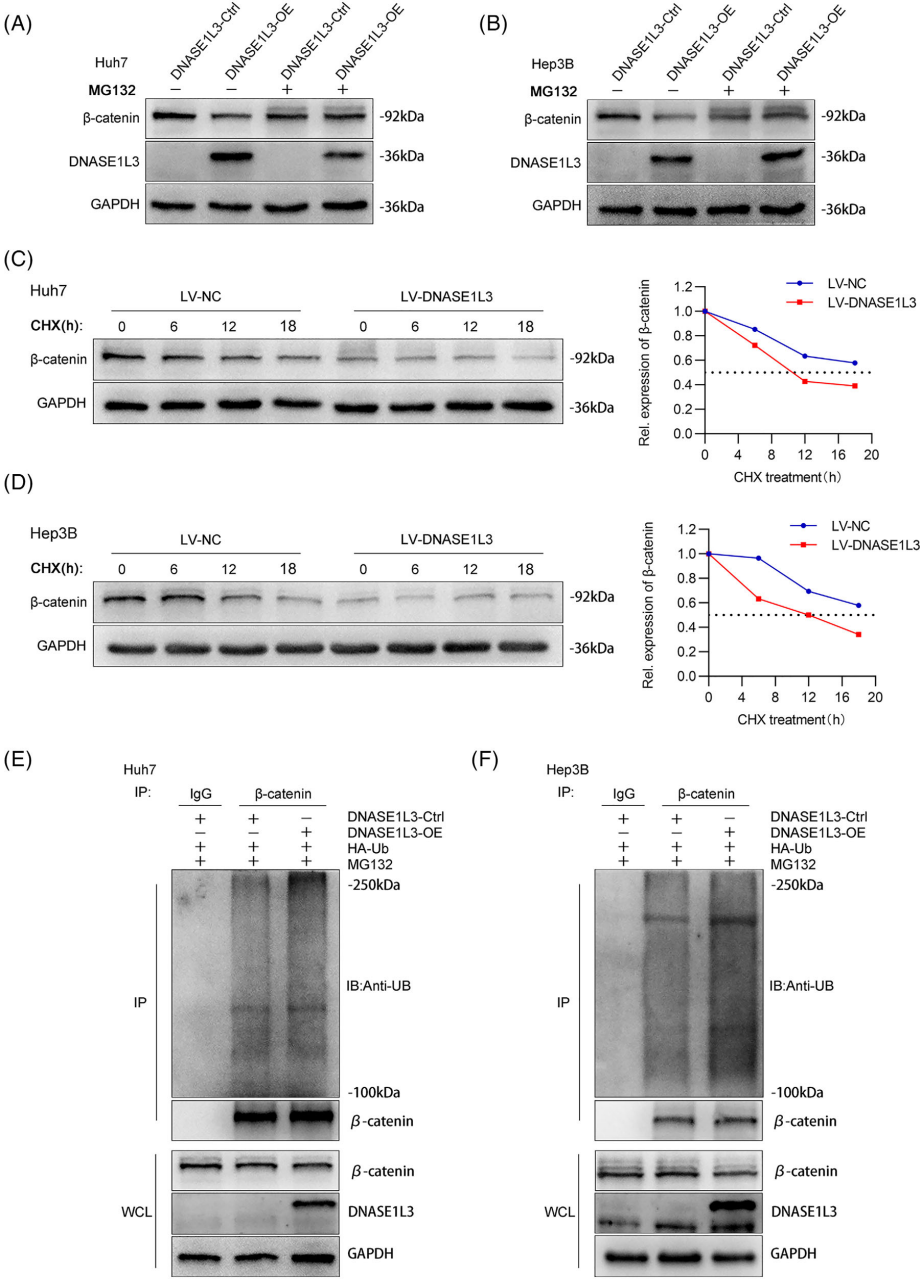
DNASE1L3 promotes β‐catenin ubiquitination and degradation. (A and B) Huh7 and Hep3B cells with or without DNASE1L3 overexpression were treated with MG132 for 8 h, and the fine cytolysis fluid was prepared. The expression of DNASE1L3 and β‐catenin protein was detected by Western blot. (C and D) Western blot showing the effect of DNASE1L3 on β‐catenin stability in Huh7 and Hep3B cells incubated with cycloheximide at different time points. (E and F) Huh7 and Hep3B cells were transfected with ubiquitin plasmid, DNASE1L3 overexpression and control plasmids. After 8 h of MG132 treatment, the cell lysate was treated with anti‐β‐catenin antibody for Co‐IP. Anti‐UB antibody was used to detect Western blot. The total cell lysate was detected by anti‐DNASE1L3, anti‐β‐catenin and anti‐GAPDH.

**FIGURE 9:**
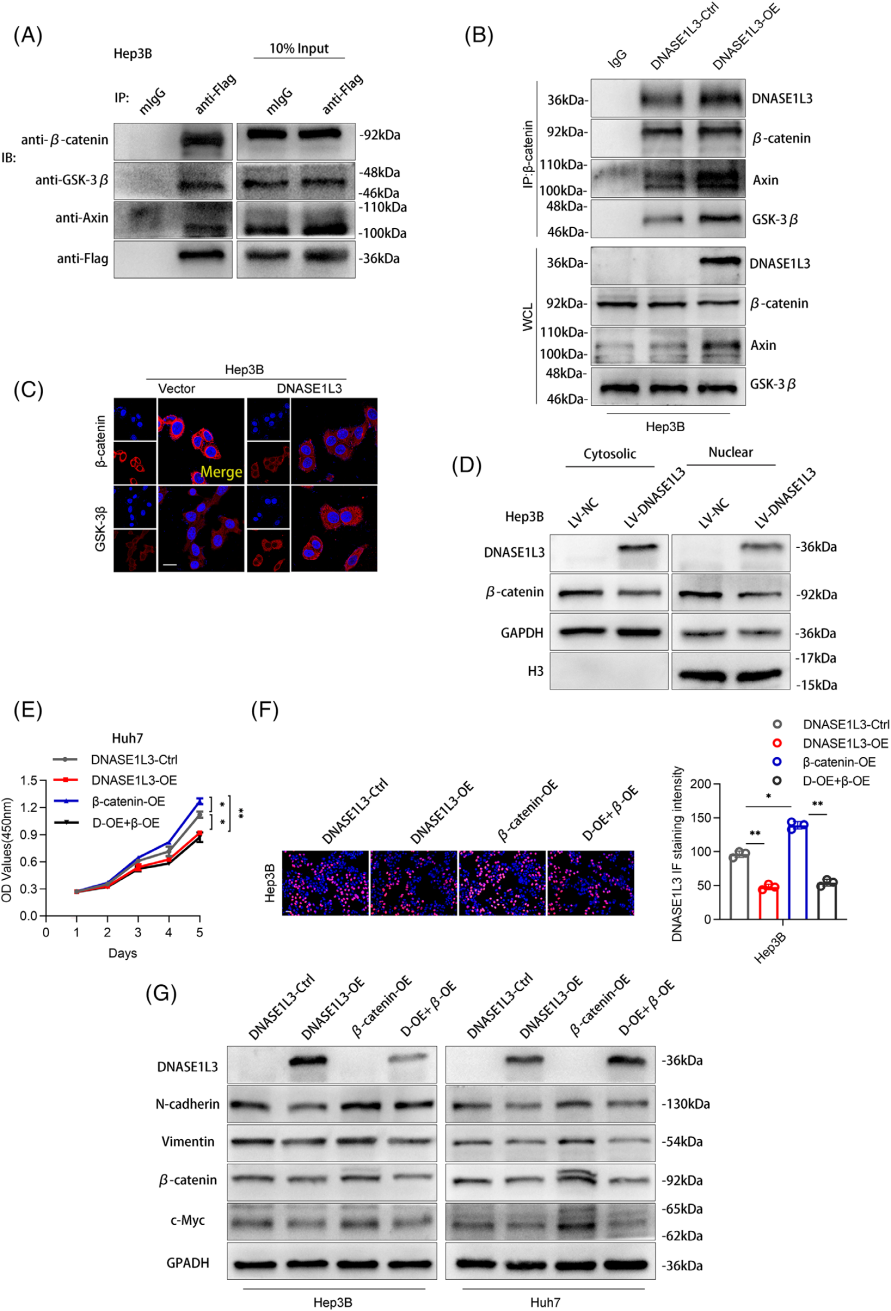
DNASE1L3 recruits more β‐catenin destruction complexes. (A) The interaction of DNASE1L3 with β‐catenin, GSK‐3 β and AXIN in Hep3B cells was determined by exogenous Co‐IP. (B) After Hep3B cells were transfected with DNASE1L3 overexpression and control plasmids, Co‐IP was performed with anti‐β‐catenin antibody, and Western blot was detected with anti‐DNASE1L3, anti‐β‐catenin, anti‐GSK‐3β and anti‐AXIN antibodies. (C) The relative fluorescence intensity of β‐catenin and GSK‐3β in DNASE1L3 overexpressed Hep3B cells was measured by exogenous cellular immunofluorescence assay. Scale bar: 20 μm. (D) The protein levels of DNASE1L3 and β‐catenin in cytoplasm and nucleus of DNASE1L3 overexpressed Hep3B cells were detected by the nucleoplasmic separation assay. (E) The cell viability of DNASE1L3‐Ctrl group, DNASE1L3‐OE group, β‐catenin‐OE group and D‐OE + β‐OE group was determined by CCK‐8 and EdU assay. Scale bar: 200 μm. (F) The protein levels of DNASE1L3, N‐cadherin, vimentin, β‐catenin, c‐Myc and GAPDH in DNASE1L3‐Ctrl group, DNASE1L3‐OE group, β‐catenin‐OE group and D‐OE + β‐OE group was detected by Western blot. Student's t‐test. Mean ± SD (*p < 0.05; **p < 0.01). D‐OE + β‐OE: overexpression of DNASE1L3 and β‐catenin by lentivirus.

We found that proteasome inhibitor MG132 can rescue the decrease in the β‐catenin protein level caused by DNASE1L3 overexpression (Figure 8A,B). β‐catenin, as a key regulator of the classical Wnt pathway, is mainly degraded by the ubiquitin‐mediated proteasome in the cytoplasm. Therefore, we speculate that DNASE1L3 can promote the degradation of β‐catenin by the proteasome pathway. In order to confirm this view, CHX chase assay was carried out to detect the change in β‐catenin half‐life following DNASE1L3 overexpression. The results showed that overexpression of DNASE1L3 decreased the half‐life of the β‐catenin protein (Fig. 8C,D). Meanwhile, β‐catenin was immunoprecipitated with a specific anti‐β‐catenin antibody, and the ubiquitination level of β‐catenin was determined by an anti‐ubiquitin antibody. As expected, overexpression of DNASE1L3 significantly increased the ubiquitination level of β‐catenin (Figure 8E,F). In order to further verify this regulatory mechanism, we speculate that DNASE1L3 may recruit more β‐catenin destruction complexes to interact with the β‐catenin protein, then regulating the protein level of β‐catenin. Co‐IP assay indicated that the protein expression of β‐catenin, GSK‐3β and Axin could be detected simultaneously when immunoprecipitating DNASE1L3 (Figure 9A). Notably, overexpression of DNASE1L3 promotes the immunoprecipitation of GSK‐3β and Axin protein (Figure 9B). Similarly, the exogenous immunofluorescence assay proved the co‐localization of DNASE1L3 and GSK‐3β protein in the cytoplasm (Figure S2B). IF analysis showed that DNASE1L3 overexpression decreased the fluorescence intensity of β‐catenin and increased the fluorescence intensity of GSK‐3β in Hep3B cells (Figure 9C). These results suggest that overexpression of DNASE1L3 promotes the degradation of β‐catenin via the ubiquitin‐proteasome pathway by recruits β‐catenin destruction complexes (GSK‐3β, Axin and β‐catenin). Moreover, the results of the nucleoplasmic separation assay showed that overexpression of DNASE1L3 decreased the levels of β‐catenin protein in cytoplasm and nucleus. To further elucidate the role of β‐catenin on the DNASE1L3 regulatory effects in HCC, the β‐catenin plasmid transfected into DNASE1L3‐overexpressing cells. CCK‐8 and EdU assays showed that DNASE1L3 upregulation impaired the cell proliferation caused by exogenous β‐catenin expression (Figure 9E,F). In addition, ectopic DNASE1L3 expression attenuated the promoting effects of β‐catenin on N‐cadherin, vimentin and c‐Myc protein levels (Figure 9G). In general, our results show that DNASE1L3 acts as a scaffold to recruit more β‐catenin destruction complexes to regulate the protein stability of β‐catenin.

### 3.6 DNASE1L3 interacts with P21 and stabilizes P21 by mediating the deubiquitination

P21, as a key cyclin‐dependent kinase (CDK) inhibitor, plays a vital role in the regulation of cell cycle. In the present study, we found that DNASE1L3 positively regulates the expression of P21 protein in the nucleus. Meanwhile, the nuclear localization sequence of DNASE1L3 itself can mediate the relocation of DNASE1L3 in the nucleus under specific circumstances. Based on the above findings, we speculate that DNASE1L3 may interacts with P21, and confirmed the links between DNASE1L3 and P21 by Co‐IP assay (Figure S3A). Interestingly, we found that DNASE1L3 regulated the expression of P21 protein without affecting its mRNA level (Figure S3B). P21 is an unstable protein under normal conditions, and the ubiquitin‐proteasome pathway is one of the main forms of its degradation. Therefore, we speculated that the regulation of P21 by DNASE1L3 occurs mainly at the post‐transcriptional level. Furthermore, the results showed that DNASE1L3‐mediated degradation of P21 was partly rescued by the proteasome inhibitor MG132 (Figure S3C). CHX chase assay was performed to show that the overexpression of DNASE1L3 prolonged the half‐life of the P21 protein (Figure S3D). These findings revealed that this process is dependent on the ubiquitin‐proteasome pathway. More importantly, P21 was immunoprecipitated with a specific anti‐P21 antibody and its ubiquitination level was examined by an anti‐ubiquitin antibody. The results showed that DNASE1L3‐overexpressing significantly reduced the ubiquitin level of P21 (Fig. S3EF). Altogether, these results suggest that DNASE1L3 can stabilize the protein level of P21 by binding to P21 and mediating its ubiquitin degradation.

## 4 DISCUSSION

The site of chromosome 3 where DNASE1L3 is located is a common site of allelic loss. Therefore, it is significantly downregulated in many cancers and is associated with certain tumour characteristics and poor prognosis.^24^, ^25^, ^26^, ^27^However, the potential molecular mechanism and clinical significance of DNASE1L3 remains to be further investigated in HCC. In this study, Based on the TCGA and GEO databases, we found that DNASE1L3 was significantly downregulated in HCC. In succeeding study, DNASE1L3 was substantially downregulated in HCC compared with paracancerous tissue. The reduction of its expression significantly promoted clinical progression and reflected the poor prognosis of HCC patients. Meanwhile, DNASE1L3 expression is an independent predictor of prognosis in HCC patients. Subsequently, we observed that DNASE1L3 repressed the proliferation, invasion and metastasis of HCC cells in vitro and in vivo. These results suggest that DNASE1L3 functions as a potential tumour suppressor in HCC.

To further explore the molecular basis of DNASE1L3 affected the progression of HCC. We identified β‐catenin as a candidate interacting protein of DNASE1L3 in Huh7 cells overexpressing DNASE1L3 by Co‐IP combined with mass spectrometry analysis. Emerging evidence has reported the β‐catenin signalling pathway plays a crucial role in the occurrence and development of HCC.^28^, ^29^, ^30^ In the classical Wnt signalling pathway, β‐catenin mainly binds to the β‐catenin destroying complex, and then GSK‐3β phosphorylates the Thr4, Ser37 and Ser33 sites of β‐catenin. Phosphorylated β‐catenin is recognized by β‐TrCP and then degraded by the 26S proteasome.^31^, ^32^, ^33^ Herein, we found that DNASE1L3 regulated the expression of β‐catenin protein without affecting its mRNA level. Furthermore, it was found that overexpression of DNASE1L3 could attenuate the stability of β‐catenin and shorten its protein half‐life using MG132 assay and CHX chase method. Moreover, the in vivo ubiquitination experiments were performed to show DNASE1L3‐overexpressing markedly enhanced the ubiquitination level of β‐catenin. Thus, we demonstrated that DNASE1L3 regulates the expression of β‐catenin through ubiquitination‐proteasome degradation manner. However, DNASE1L3 does not have the ability to transmit ubiquitin. Interestingly, we had found that DNASE1L3 can recruit β‐catenin destruction complex, enhance the interaction between β‐catenin destruction complex and β‐catenin and impair the stability of β‐catenin. Notably, we verified that DNASE1L3 can inhibit the entry of β‐catenin into the nucleus using nucleocytoplasmic separation assay, and obstruct the expression levels of downstream targets (c‐Myc, P21 and P27), eventually regulated β‐catenin‐related tumour properties and EMT signals.

Previous reports indicated the C‐terminal helical domain of DNASE1L3 contains a nuclear localization sequence, which allows it to translocate from the endoplasmic reticulum to the nucleus during apoptosis. In our study, DNASE1L3 has a positively effect on the expression of P21 protein was observed in HCC cells. P21 is a key member of the cyclin‐dependent kinase inhibitor family that inhibits the cell cycle by directly binding cyclins and CDKs.^34^, ^35^ Previous studies have found that P21 expression is reduced or absent in various cancers, such as HCC.^36^, ^37^ Overexpression of P21 can lead to cell cycle arrest in G1 phase, those findings indicated that P21 acts as a tumour suppressor in the nucleus and affects cell proliferation. P21 is an unstable protein with a relatively short half‐life and is mainly degraded by the ubiquitin‐proteasome pathway.^38^, ^39^, ^40^ In this study, DNASE1L3‐overexpressing was found to inhibit the ubiquitination and degradation of P21, stabilize its protein expression, thereby causing G1 phase arrest and lessening cell proliferation. However, further studies are required to fully understand the potential mechanism of DNASE1L3 enhance P21 deubiquitination.

In summary, our findings confirmed the negatively regulatory effect of DNASE1L3 on HCC. DNASE1L3 can physically bind β‐catenin and inhibit its nuclear translocation, thus, inhibiting the proliferation and metastasis of HCC cells. In mechanism, DNASE1L3 promotes ubiquitination‐related degradation of β‐ catenin and inhibits downstream targets c‐Myc, P21 and P27 by recruiting β‐catenin destruction complex, thus controlling the cell cycle and EMT signal. In addition, targeting DNASE1L3 may be an effective strategy for HCC treatment. Diagnostic value of serum DNASE1L3 in hepatitis B virus‐related hepatocellular carcinoma.

## AUTHOR CONTRIBUTIONS

Shi Zuo planned the experiments and revised the manuscript. Bo Li, Wei‐Wei Yan, Bin Gong performed the experiments and prepared a draft of the manuscript. Kun Cao, Ye‐Wei Zhang, Rui Zhao and Yi‐Heng Jiang performed the statistical analysis. Bo Li, Yu‐Zhen Ge conceived the project and edited the manuscript. Chao Li and Bo Li discussed the results. All the authors read and approved the final manuscript.

## CONFLICT OF INTEREST

The authors declare no conflict of interests.

## Supporting information


**Figure S1** The expression of DNASE1L3 and the efficiency of plasmid and virus. The protein level of DNASE1L3 in 12 pairs of HCC and paracancerous tissues was analyzed by Western blot. (B and C) The mRNA and protein levels of DNASE1L3 in hepatoma cell lines (HCCLM3, Hep3B, PLC/PRF5, Huh7, MHCC97H, Hep3B and HepG2) were determined by RT‐qPCR and Western blot, the normal liver cell LO2 was used as control. (D) Univariate or multivariate COX regression analysis was used. The results show that DNASE1L3 may be an independent indicator of disease‐free survival in patients with HCC. (E) The GFP images of Hep3B and HCCLM3 cells infected with LV‐NC and LV‐DNASE1L3 were observed by fluorescence microscope. Scale bar: 200 μm. (F) The efficiency of infection was determined by Western blot in Hep3B and Huh7 cells infected with lentivirus. (G) The efficiency of transient knockdown after DNASE1L3 overexpression was detected by RT‐qPCR. (H) The representative peptides of DNASE1L3 and β‐catenin in MS results. Student's *t* test. Mean ± SD (*p < 0.05; **p < 0.01).

**Figure S2** Effects of DNASE1L3 on β‐catenin and its colocalization with GSK‐3β. (A) Different gradients of DNASE1L3 overexpression and its control plasmids were introduced into 293 T cells, and the expression levels of DNASE1L3 and β‐catenin were measured by Western blot. (B) The interaction between DNASE1L3 and GSK‐3β in Hep3B cells was determined by exogenous cellular immunofluorescence assay. Scale bar: 20 μm.

**Figure S3** DNASE1L3 promotes the deubiquitination of P21. (A) The interaction between DNASE1L3 and P21 in Hep3B cells was determined by exogenous Co‐IP assay. (B) The mRNA level of P21 was detected by RT‐qPCR after DNASE1L3 overexpression or knockdown. (C) Hep3B cells with or without DNASE1L3 overexpression were treated with MG132 for 8 h, and the cell lysate was prepared. The expression of DNASE1L3 and P21 protein was detected by Western blot. (D) Western blot showing the effect of DNASE1L3 on P21 stability in Hep3B cells incubated with cycloheximide at different time points. (E and F) Huh7 and Hep3B cells were transfected with ubiquitin plasmid, DNASE1L3 overexpression and control plasmids. After being treated with MG132 for 8 h, the cell lysate was Co‐IP with anti‐P21 antibody. Anti‐UB antibody was used to detect Western blot. The total cell lysate was detected by Western blot with anti‐DNASE1L3, anti‐P21 and anti‐GAPDH. Student's *t* test. Mean ± SD (*p < 0.05; **p < 0.01).

**Table S1.** Correlation of DANSE1L3 expression with clinicopathological characteristics of patients with HCC.
**Table S2**. The sequences used in this study.
**Table S3**. The primers used in this study.
**Table S4**. A list of antibodies used for Western blot.

## Data Availability

The data used to support the findings of this study are included within the article.
