# Supplementary figures and images for: DNASE1L3 inhibits proliferation, invasion and metastasis of hepatocellular carcinoma by interacting with β‐catenin to promote its ubiquitin degradation pathway
Source: Cell Prolif. 2022 Jun 24;55(9):e13273. doi: 10.1111/cpr.13273 (PMC9436914; doi:10.1111/cpr.13273)

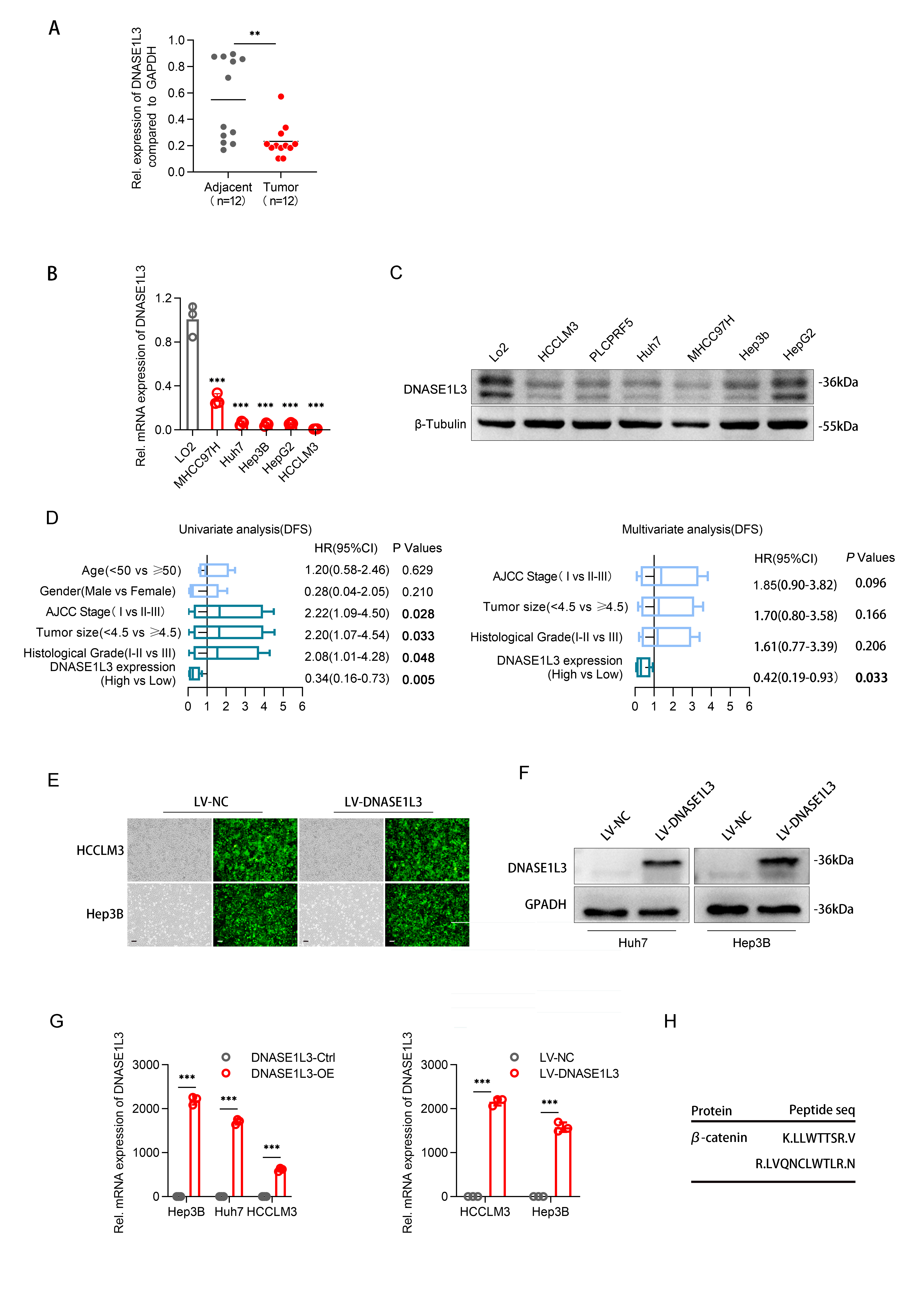

Supplement: Supplementary file 1 — Figure S1 The expression of DNASE1L3 and the efficiency of plasmid and virus. The protein level of DNASE1L3 in 12 pairs of HCC and paracancerous tissues was analyzed by Western blot. (B and C) The mRNA and protein levels of DNASE1L3 in hepatoma cell lines (HCCLM3, Hep3B, PLC/PRF5, Huh7, MHCC97H, Hep3B and HepG2) were determined by RT‐qPCR and Western blot, the normal liver cell LO2 was used as control. (D) Univariate or multivariate COX regression analysis was used. The results show that DNASE1L3 may be an independent indicator of disease‐free survival in patients with HCC. (E) The GFP images of Hep3B and HCCLM3 cells infected with LV‐NC and LV‐DNASE1L3 were observed by fluorescence microscope. Scale bar: 200 μm. (F) The efficiency of infection was determined by Western blot in Hep3B and Huh7 cells infected with lentivirus. (G) The efficiency of transient knockdown after DNASE1L3 overexpression was detected by RT‐qPCR. (H) The representative peptides of DNASE1L3 and β‐catenin in MS results. Student's t test. Mean ± SD (*p < 0.05; **p < 0.01). [file CPR-55-e13273-s004.png]

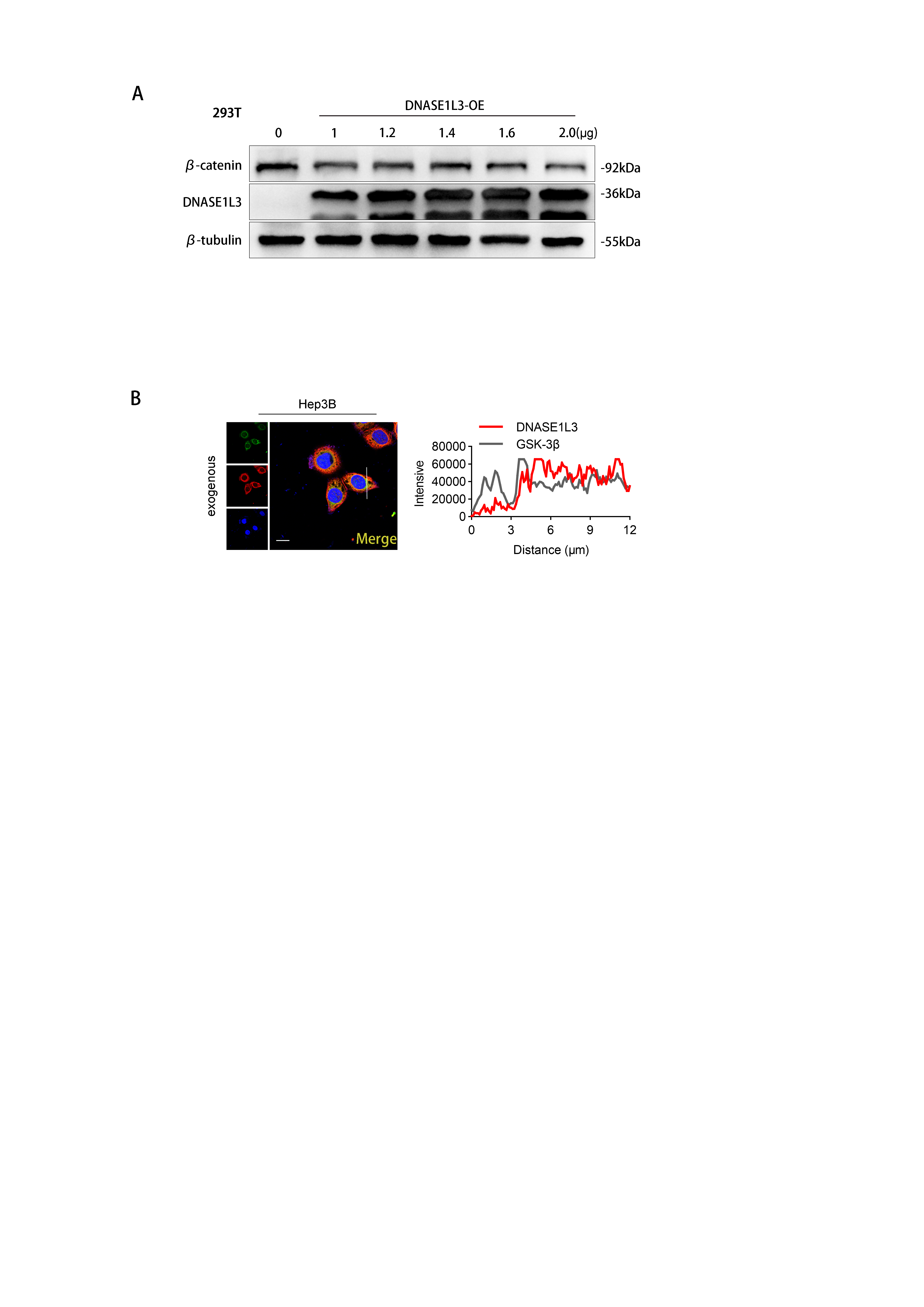

Supplement: Supplementary file 2 — Figure S2 Effects of DNASE1L3 on β‐catenin and its colocalization with GSK‐3β. (A) Different gradients of DNASE1L3 overexpression and its control plasmids were introduced into 293 T cells, and the expression levels of DNASE1L3 and β‐catenin were measured by Western blot. (B) The interaction between DNASE1L3 and GSK‐3β in Hep3B cells was determined by exogenous cellular immunofluorescence assay. Scale bar: 20 μm. [file CPR-55-e13273-s003.png]

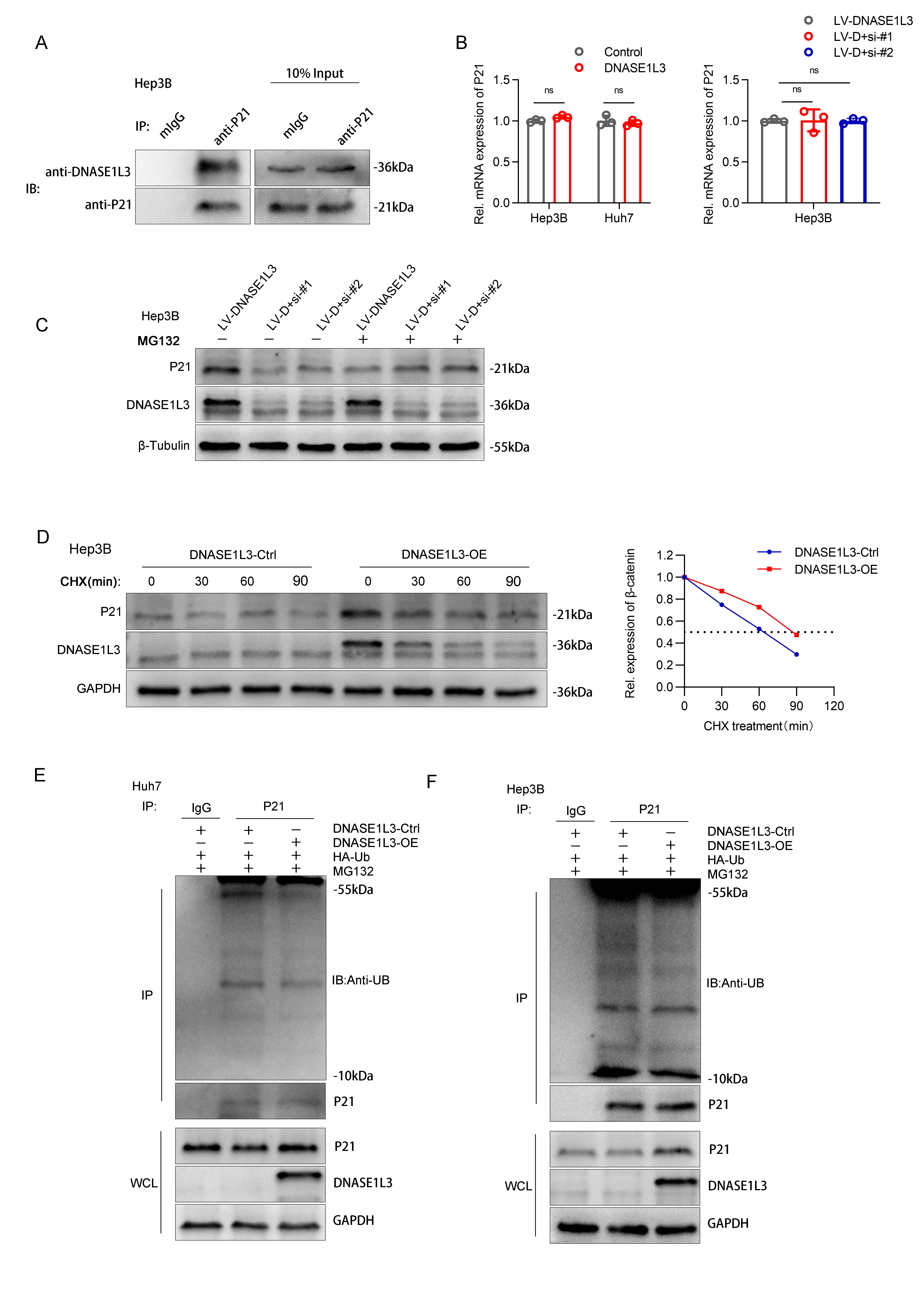

Supplement: Supplementary file 3 — Figure S3 DNASE1L3 promotes the deubiquitination of P21. (A) The interaction between DNASE1L3 and P21 in Hep3B cells was determined by exogenous Co‐IP assay. (B) The mRNA level of P21 was detected by RT‐qPCR after DNASE1L3 overexpression or knockdown. (C) Hep3B cells with or without DNASE1L3 overexpression were treated with MG132 for 8 h, and the cell lysate was prepared. The expression of DNASE1L3 and P21 protein was detected by Western blot. (D) Western blot showing the effect of DNASE1L3 on P21 stability in Hep3B cells incubated with cycloheximide at different time points. (E and F) Huh7 and Hep3B cells were transfected with ubiquitin plasmid, DNASE1L3 overexpression and control plasmids. After being treated with MG132 for 8 h, the cell lysate was Co‐IP with anti‐P21 antibody. Anti‐UB antibody was used to detect Western blot. The total cell lysate was detected by Western blot with anti‐DNASE1L3, anti‐P21 and anti‐GAPDH. Student's t test. Mean ± SD (*p < 0.05; **p < 0.01). [file CPR-55-e13273-s001.png]
