# Supplementary material for: DNASE1L3 inhibits proliferation, invasion and metastasis of hepatocellular carcinoma by interacting with β‐catenin to promote its ubiquitin degradation pathway
Source: Cell Prolif. 2022 Jun 24;55(9):e13273. doi: 10.1111/cpr.13273 (PMC9436914; doi:10.1111/cpr.13273)
Supplement: Supplementary file 4 — Table S1. Correlation of DANSE1L3 expression with clinicopathological characteristics of patients with HCC. Table S2. The sequences used in this study. Table S3. The primers used in this study. Table S4. A list of antibodies used for Western blot. [file CPR-55-e13273-s002.docx]

**Supplementary Table Ⅰ.**

**Correlation of DANSE1L3 expression with clinicopathological characteristics of patients with HCC.**

| **Characteristics** | **n** | **DNASE1L3 expression** | | ***P*** |
| --- | --- | --- | --- | --- |
|  |  | **Low** | **High** |  |
| **Gender** |  |  |  |  |
| Male | 80 | 40(50.00%) | 40(50.00%) | 1.000 |
| Female | 10 | 5 (50.00%) | 5 (50.00%) |  |
| **Age** |  |  |  |  |
| <50 | 37 | 19(51.35%) | 18(48.65%) | 0.830 |
| ≥50 | 53 | 26(49.06%) | 27(50.94%) |  |
| **AJCC Stage** |  |  |  |  |
| I | 63 | 27(42.86%) | 36(57.14%) | **0.038** |
| II-III | 27 | 18(66.67%) | 9(33.33%) |  |
| **Tumor size(cm)** |  |  |  |  |
| <4.5 | 48 | 19(39.58%) | 29(60.42%) | **0.035** |
| ≥4.5 | 42 | 26(61.90%) | 16(38.10%) |  |
| **Vital Status** |  |  |  |  |
| Alive | 58 | 24(41.38%) | 34(58.62%) | **0.028** |
| Dead | 32 | 21(65.62%) | 11(34.38%) |  |
| **Recurrence** |  |  |  |  |
| No | 41 | 15(36.59%) | 26(63.41%) | **0.020** |
| Yes | 49 | 30(61.22%) | 19(38.78%) |  |
| **Cirrhosis** |  |  |  |  |
| Absent | 9 | 5 (55.56%) | 4 (44.44%) | 0.699 |
| Present | 80 | 39(48.75%) | 41(51.25%) |  |
| **HBsAg** |  |  |  |  |
| Absent | 19 | 10(52.63%) | 9 (47.37%) | 0.754 |
| Present | 70 | 34(48.57%) | 36(51.43%) |  |
| **ALT level(U/L)** |  |  |  |  |
| <50 | 60 | 29(48.33%) | 31(51.67%) | 0.764 |
| ≥50 | 29 | 15(51.72%) | 14(48.28%) |  |
| **AFP level(ng/L)** |  |  |  |  |
| <400 | 57 | 27(47.37%) | 30(52.63%) | 0.602 |
| ≥400 | 32 | 17(53.12%) | 15(46.88%) |  |
| **Histological Grade** |  |  |  |  |
| I-II | 66 | 29(43.94%) | 37(56.06%) | 0.057 |
| III | 24 | 16(66.67%) | 8 (33.33%) |  |

**Supplementary Table Ⅱ.**

**The sequences used in this study.**

| Gene | No. | Target sequences |
| --- | --- | --- |
| DNASE1L3 | stB0003772A | GTGACATCATACTCGTGAT |
|  | stB0003772B | CGACCACTTTCCAGTTGAA |
|  | stB0003772C | CAGAGACATCCGTTAAGGA |

**Supplementary Table Ⅲ.**

**The primers used in this study.**

| Primers name |  | Sequence (5’-3’ ) |
| --- | --- | --- |
| DNASE1L3 | Forward | TGCTCCTTCAACGTCAGGTC |
|  | Reverse | CCAGCTTTTCCCTGTTCAGC |
| β-catenin | Forward | GGAAGGTCTGAGGAGCAGC |
|  | Reverse | TCCAACTCCATCAAATCAGCTTG |
| P21 | Forward | TGCCGAAGTCAGTTCCTTGT |
|  | Reverse | GTTCTGACATGGCGCCTCC |
| GAPDH | Forward | TGCACCACCAACTGCTTAGC |
|  | Reverse | GGCATGGACTGTGGTCATGAG |

**Supplementary Table Ⅳ.**

**A list of antibodies used for Western blot.**

| Antibodies | Company | Cat.No | Mol weight (kDa) | Dilution |
| --- | --- | --- | --- | --- |
| DNASE1L3 | Abcam | Ab152118 | 36 | 1:1000(WB)  1:100(IF) |
| β-catenin | Proteintech | 51067-2-AP | 92 | 1:1000(WB)  4.0µg(IP)  1:100(IF) |
| GSK-3β | Proteintech | 22104-1-AP | 46-48 | 1:1000(WB)  1:100(IF) |
| Axin | Proteintech | 16541-1-AP | 100-110 | 1:1000(WB) |
| c-Myc | Proteintech | 10828-1-AP | 62-65 | 1:1000(WB) |
| P21 | Proteintech | 10355-1-AP | 21 | 1:1000(WB)  4.0µg(IP) |
| P27 | Proteintech | 25614-1-AP | 27 | 1:1000(WB) |
| E-cadherin | Proteintech | 60335-1-Ig | 120 | 1:1000(WB) |
| N-cadherin | Proteintech | 66219-1-Ig | 130 | 1:1000(WB) |
| Vimentin | Proteintech | 10366-1-AP | 54 | 1:1000(WB) |
| Flag | Sigma | F1804 | - | 1:1000(WB)  4.0µg(IP)  1:100(IF) |
| Ubiquitin | Proteintech | 10201-2-AP | - | 1:1000(WB) |
| Goat Anti-Mouse IgG H&L (Alexa Fluor® 647) | Abcam | ab150115 | - | 1:200(IF) |
| Goat Anti-Rabbit IgG H&L (Alexa Fluor® 555) | Abcam | ab150078 | - | 1:200(IF) |
| GAPDH | Bioworld | pAb AP0063 | 36 | 1:10000(WB) |
| β-tubulin | Bioworld | pAb AP0064 | 55 | 1:5000(WB) |
